# Effect of Heat Pasteurization and Sterilization on Milk Safety, Composition, Sensory Properties, and Nutritional Quality

**DOI:** 10.3390/foods14081342

**Published:** 2025-04-14

**Authors:** Ahmad Rabbani, Mutamed Ayyash, Crystal D. C. D’Costa, Gang Chen, Yajun Xu, Afaf Kamal-Eldin

**Affiliations:** 1 Department of Food Science, College of Agriculture and Veterinary Medicine, United Arab Emirates University, Al Ain P.O. Box 15551, United Arab Emirates; 202090507@uaeu.ac.ae (A.R.); mutamed.ayyash@uaeu.ac.ae (M.A.);; 2 Key Laboratory of Geriatric Nutrition and Health (Beijing Technology and Business University), Ministry of Education, 100048, China; gang.chen@btbu.edu.cn; 3 Beijing Key Laboratory of Toxicological Research and Risk Assessment for Food Safety, Department of Nutrition and Food Hygiene, School of Public Health, Peking University, No. 38 Xueyuan Road, Beijing 100083, China; xuyajun@bjmu.edu.cn; 4 National Water and Energy Center, United Arab Emirates University, Al Ain P.O. Box 15551, United Arab Emirates

**Keywords:** pasteurization, sterilization, milk safety, vitamins, minerals, nutritional quality

## Abstract

Milk pasteurization and sterilization by heat treatment have an exciting history, which followed steady steps. The main aim of these treatments is to extend the shelf life of milk by destroying pathogenic and milk spoilage bacteria. With developments in pasteurization techniques, the assurance of milk safety, and extended shelf life, pasteurized bovine milk has become a staple food, especially in Western diets. However, some concerns have recently been raised about the effect of pasteurization on the sensory properties and nutritional quality of milk, and alternative methods, such as high-pressure processing, are being investigated. The primary purpose of milk pasteurization and sterilization is summarized in this review article. The associated changes that affect the compositional, sensory, and nutritional quality of milk are discussed, with particular emphasis on protein structure and function. The review is concluded by considering alternative methods, their advantages and limitations, along with future prospects.

## 1. Introduction

**Figure 1:**
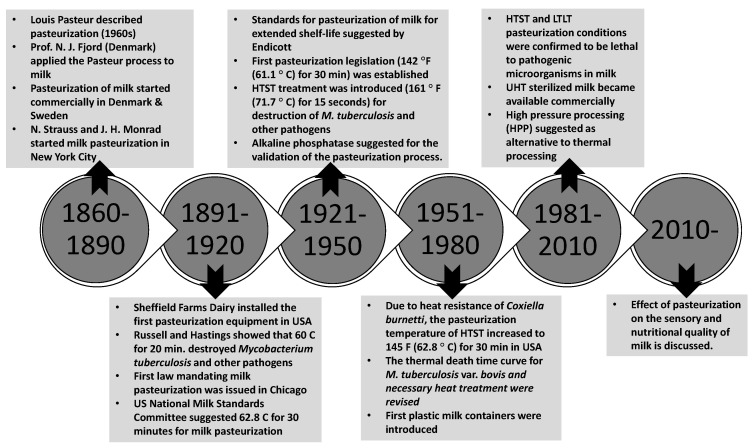
A summary of historical developments in the conceptualization and application of milk pasteurization [2,6].

After the French revolution, Nicolas Appert made a breakthrough in food preservation in 1809 by describing the appertization process that combines canning and heating [1]. Louis Pasteur later set a theoretical framework for how heat treatment preserves foods by destroying spoilage microorganisms. In 1863, Pasteur solved the spoilage problems of wine and beer by carrying out heating at temperatures and times sufficiently long to inactivate spoilage microorganisms in a process later named pasteurization [2]. Milk pasteurization on a commercial scale started in Denmark and Sweden after Strauss and Monrad led a campaign promoting pasteurization throughout the USA in 1889 [2]. Koplik [3] found that the consumption of raw milk contributed to pathogen transmission from animals to humans. In the same year, milk pasteurization became common in Denmark to reduce the risk of spreading tuberculosis [4]. Sheffield Dairy Farms installed the first pasteurization equipment in Bloomfield, New Jersey, USA, in 1891. Soxhlet suggested that milk used to feed infants must be heated for public health reasons [2]. The first law mandating milk pasteurization was issued in Chicago in 1909. Thereafter, pasteurization became standard practice in the commercial milk industry [4]. A narrative history of the conceptualization and implementation of milk pasteurization is provided in Figure 1.

Several pasteurization techniques have been developed with variable effects on milk quality and shelf life. Due to its increased shelf life, pasteurized milk has become a staple food and an integral part of the diet, especially in the West. However, in recent years, there has been some resurgence in the consumption of raw milk, largely driven by the belief that unprocessed milk offers better health benefits than heat-treated milk. This perception has led to an increase in the consumption of raw milk by sensitive groups, including infants, the elderly, and immunocompromised individuals, as well as those adhering to specific dietary preferences [5]. This trend has ignited ongoing debates about the safety and nutritional merits of raw and pasteurized milk for direct consumption. While “natural” food products are often viewed favorably by the public, scientific evidence does not inherently equate naturalness with safety, healthiness, or taste. Between 2007 and 2012, various milk-borne outbreaks were reported in the European Union, with raw milk being implicated in several cases. In response, institutions such as the European Food Safety Authority (EFSA), the U.S. Food and Drug Administration (FDA) [6], and the Centers for Disease Control and Prevention (CDC) have issued evaluations and advisories highlighting the potential risks associated with raw milk consumption [7].

Simultaneously, interest in the sensory properties and health implications of milk consumption has increased, with some believing that the nutritional and sensory values of milk are adversely affected by pasteurization. Thus, alternative methods, such as high-pressure processing (HPP), power ultrasonics (PU), pulsed electric fields (PEFs) and microfiltration (MF), are being investigated [8,9,10,11]. This review article aims to summarize the primary purpose of milk pasteurization and discuss the associated changes that may affect not only the safety but also the compositional, sensory, and nutritional quality of milk. Specifically, particular emphasis is placed on the effects of milk pasteurization on protein structure and function. Finally, alternative methods to pasteurization, their advantages, and their limitations, along with future prospects, are discussed in the conclusion of this article.

## 2. Pasteurization and Sterilization Processes and Bacterial Destruction

Dr. Richard Seligman described pasteurization in 1923 as an energy-efficient process by which microorganisms were destroyed, with minor damage to the physicochemical properties of milk [12]. According to the FAO/WHO (2004) [13], pasteurization can be defined as a heat treatment designed to reduce the number of pathogenic microorganisms found in milk and liquid milk products. The quality of raw milk is determined by its bacterial count, which depends on both the health of the animal and potential contamination after milking. Thus, the final quality of the milk is significantly influenced by the somatic cell count (SCC), which reflects the immune response of the animal, particularly in cases of mastitis (udder inflammation). Enzymes associated with a high SCC in milk cause protein and fat degradation and may produce off-flavors during refrigerated storage. Healthy cows generally have SCC < 50,000 cells/mL of milk. In contrast, SCC in bulk tank milk may exceed 200,000 cells/mL due to the contribution of samples of high SCC from a few animals in the herd [14,15].

Milk pasteurization aims to serve two primary purposes, i.e., killing pathogenic bacteria and reducing the number of live spoilage bacteria, leading to the prolongation of the shelf life of the milk. After establishing the importance of pasteurization on milk safety, scientists focused on the details of pasteurization and sterilization processes. The initial pasteurization conditions, known as flash pasteurization, in which the milk was heated to up to ~80 °C, continued to be used until the 1960s. Enright [16] showed that older pasteurization processes were inadequate to inactivate *Coxiella burnetti*, which causes Q-fever in humans, and suggested new pasteurization conditions, i.e., heating at 62.8 °C for 30 min for batch processes and heating at 71.7 °C for 1 s for continuous processes. Different heat treatments, such as thermal treatment (65 °C for 15 s), low-temperature long-time pasteurization (LTLT, 65 °C for 30 min), high-temperature short-time pasteurization (HTST, 72 °C for 15 s), extended shelf life pasteurization (120–130 °C for 1–4 s), ultra-high-temperature sterilization (UHT, 136–145 °C for 2–8 s), in-container or vat sterilization (112 °C for 15 min), and innovative steam injection (ISI) treatments, are currently available (Table 1). These processes target variable microbial species and result in milk with different shelf lives [17]. Thermal treatments destroy heat-sensitive spoilage bacteria; pathogenic bacteria are mainly eliminated by pasteurization, while sterilization kills all bacteria and spores [2,18,19].

**Figure 2:**
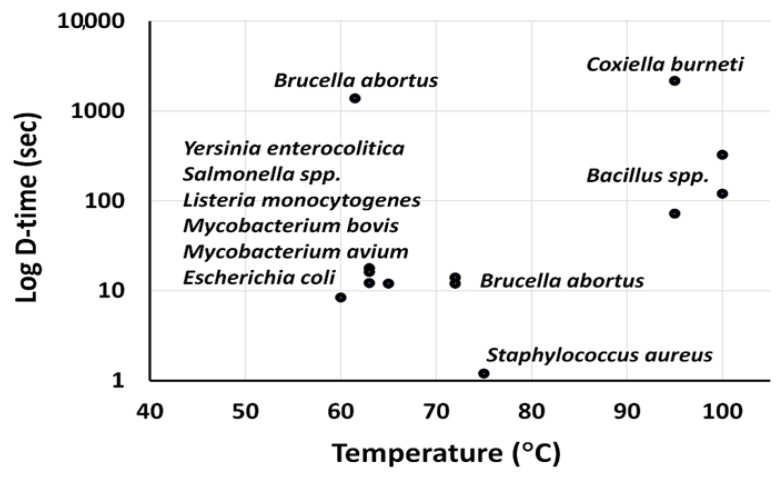
The relationship between pasteurization temperature and log D-time (the time required to reduce the number of microorganisms in one log cycle) [29].

The primary goal of pasteurization conditions is to destroy *Mycobacterium tuberculosis* and *C. burnett* (i.e., the most temperature-resistant milk pathogens). The effects of certain temperature–time combinations on different pathogenic species are described in Figure 2. About 99.9% of pathogens are killed by pasteurization through HTST, and viable *Mycobacterium avium* populations are effectively reduced. All vegetative pathogens, such as human pathogenic *Escherichia coli*, *Listeria* spp., *Salmonella* spp., *Campylobacter jejuni*, and *Clostridium botulinum*, are destroyed by HTST pasteurization. However, heat-resistant spores of *Bacillus cereus* or *C. botulinum* are not destroyed by pasteurization. Instead, the germination of these spores can be induced while refrigerating pasteurized milk. Vegetative and most sporulating pathogens, especially *C. botulinum* and *B. cereus,* but not the spores of some extreme heat-resistant nonpathogens, such as *Bacillus thermodurans*, are destroyed by higher-temperature treatments (e.g., UHT, sterilization, and ISI). The efficiency of pasteurization processes can be determined by measuring the activity of alkaline phosphatase, which is a natural enzyme in mammalian kinds of milk with higher thermal tolerance than the most heat-resistant, non-spore-forming pathogens commonly found in milk [20,21]. Positive phosphatase activity indicates that the milk has not been sufficiently pasteurized or has been contaminated with raw milk or bacteria after processing [22].

**Table 1:** Different heat treatments used in the milk and dairy industries [23].

Heat Treatments	Temperature–Time Combination Required	Time	Pathogens Destroyed
Thermization	57–68 °C	5 s–30 min	Non-spore-forming pathogens and psychrotropic spoilage bacteria
Flash pasteurization	72–80 °C	15–30 s	Non-spore-forming pathogens and psychrotropic spoilage bacteria
Extended shelf life pasteurization (ESLP)	125–140 °C	1–10 s	Psychrotropic, mesophilic, and non-spore-forming bacteria
HTST	72–74 °C	15–20 s	*Coxiella burnetii*, the most heat-resistant pathogen in raw milk
Ultra-high-temperature (UHT) indirect heating	130–145 °C	5–20 s	*Clostridium Botulinum* and target *Coxiella burnetii;* bacterial endospore
Ultra-high-temperature (UHT) direct heating	142–150 °C	2–6 s	Heat-resistant spore formers without excessive chemical damage
Sterilization	110–120 °C or 125 °C	10–20 min 5 min	All non-spore-forming bacteria except heat-resistant spore-forming bacteria
Innovative steam injection (ISI)	160–180 °C	0.1 s	Heat-resistant spores

The spoilage of pasteurized milk is caused by post-pasteurization contamination with Gram-negative psychrotrophic bacteria, such as *Enterobacter*, *Serratia*, *Hafnia*, *Citrobacter*, *Pseudomonas*, *Alcaligenes*, and *Flavobacterium* [24]. The destruction of lactic acid bacteria by pasteurization increases milk’s shelf life. However, it may also lead to unintended consequences, such as the increased growth of bacterial spores (e.g., *Bacillus* spores) and vegetative bacteria that survive pasteurization in the absence of lactic acid bacteria. Thus, when implementing the pasteurization of milk, there is a need for septic packaging to prevent recontamination post-pasteurization. Recyclable glass bottles were initially used until Ruben Rausing introduced paperboard containers in Sweden [25]. The Tetra Classic triangular (1951) was created, followed by Tetra Brick (1963), and finally, the Tetrapack packages currently used [26]. It has been proven in follow-up studies that milk pasteurization has contributed enormously to the public health and safety of populations worldwide [27]. However, the recent identification of heat-resistant microorganisms of public health significance, such as *Listeria monocytogenes* and *Mycobacterium avium* subsp. *paratuberculosis*, may question the adequacy of pasteurization heat treatment [28].

## 3. Effects of Pasteurization and Sterilization on Milk Safety

Interest and popularity in the consumption of raw unpasteurized milk are increasing day by day as it is suggested in some reports that healthy microflora and bioactive components present in milk are also destroyed by pasteurization. The main reasons used to advocate the consumption of raw milk are its enhanced nutritional value, health benefits, overall quality, and better taste [27]. However, these claims have not been substantiated due to a lack of scientific evidence and the high cost of the required investigations. Instead, various epidemiological studies have confirmed that the contamination of raw milk by various pathogens causes the transmission of infectious diseases in humans [9,30,31]. The prevalence level of different pathogens available in raw milk has been reported in various research studies; for example, bacteria like *L. monocytogenes* and *C. jejuni* are found in unpasteurized milk at a prevalence level of about 12–13% [32]. The prevalence of pathogens in milk is influenced by many factors, including milking facilities, farm hygiene, different seasons, farm management practices, the type of utensils used to store milk, and the number of animals available on the farm [33]. One of the crucial sources of foodborne pathogens includes dairy farms, and the level of contamination in raw milk depends to a large extent upon the milking and farming practices used. Pathogens can contaminate raw milk even if it appears to be of acceptable quality and from healthy animals [34]. The possible mechanisms by which pathogens can contaminate raw milk are systematic infections, udder infections, feed contamination, and human transmission during milking.

Various foodborne pathogens causing outbreaks are present in raw milk, like *Salmonella* spp., *Campylobacter*, and some verocytotoxin-producing human pathogens like *E. coli*, *Y. enterocolitis*, *E. coli* O157:H7, and *L. monocytogenes* [35]. Nearly 2–6% of foodborne bacterial diseases in many industrialized countries are caused by pathogens in raw unpasteurized milk. Human pathogens found in raw milk are responsible for causing foodborne illnesses and outbreaks. When a high number of *Staphylococcus aureus* is present in raw milk due to contamination, it produces an enterotoxin, which is harmful to humans. Listeriosis is a condition that occurs when there is a high infectious load of *L. monocytogenes* in milk [36]. Common symptoms of milk-borne infections include abdominal cramps, diarrhea, nausea, vomiting, fever, etc. More severe symptoms, such as hemolytic uremic syndrome caused by *E. coli* O157:H7 and Guillain-Barré syndrome caused by *Campylobacter* spp., can be seen in people with severe illnesses. These symptoms may also result in chronic problems such as arthritis or, in some cases, even death.

Raw milk has been identified as a frequent source of various foodborne outbreaks and illnesses (Table 2). Statistical analysis data for milk-related human outbreaks in the United States have been reported and reviewed [37]. According to these data, consuming raw milk products was responsible for 121 outbreaks, which resulted in 1571 confirmed cases, including two deaths and 202 hospitalizations. A high number of outbreaks (55) were reported in 21 states where the sale of raw milk was allowed compared to a smaller number of cases and outbreaks in states where raw milk sale was banned and prohibited [37]. The outbreaks, illnesses, and health issues reported due to the consumption of raw milk reflect only a small proportion of the actual numbers [38,39]. For example, between 2001 and 2010, it was shown in data from Minnesota that 3.7% of patients acquired sporadic infections due to the consumption of raw unpasteurized milk [40]. In Minnesota, it was estimated that 20,500 patients (17%) had sporadic infection and enteric pathogen infection due to the consumption of raw milk. This resulted in a raw-milk-associated outbreak and illnesses. Children under the age of five were adversely affected, and it was observed that 76% of them were given raw unpasteurized milk from their farms [40].

*S. aureus* is the primary pathogen associated with contagious mastitis, which occurs at 24.4–37% in bulk tank milk samples. Contamination by coagulase-negative *Staphylococcus* and *Streptococcus* spp. was also shown in colostrum and bulk tank milk. The prevalence rates for *S. aureus* and *S. agalactiae* isolation were 31% and 10%, respectively [41]. Sixteen enterotoxin genes (seg–seq, sea–see), along with the toxic shock syndrome toxin gene (tsst-1), isolated from *S. aureus* in milk from mastitis-affected cows, were compared for their prevalence. A total of 73 out of 78 *S. aureus* isolates were positive for one or more enterotoxin genes. Along with the enterotoxin genes sed and tsst-1, some new *Staphylococcus* enterotoxin genes (e.g., sem, sen, and sei) were also reported in most *S. aureus* isolates. The high epidemiological prevalence of enterotoxin and *S. aureus* in raw milk is a concern since this species is a common pathogen isolated from raw milk and is responsible for foodborne infections and outbreaks [42].

**Table 2:** Outbreaks related to raw milk and raw milk products from available epidemiological data.

Year	Pathogen	State	Outbreak Information	Reference
2015	*Campylobacter jejuni*	Italy	*Campylobacter jejuni* contamination of raw milk across several Italian regions was estimated to cause between 230,776 and 301,785 cases per year (D–R I model) and up to 5.25 million cases per year under worst-case assumptions (D–R II model) depending on storage conditions.	[43]
2014	*Staphylococcus aureus*	Italy	There were no reported outbreaks, but an estimated 485 servings per year contained ≥20 ng enterotoxin A.	[44]
2013	*Campylobacter* spp. *L. monocytogenes* *Salmonella* spp.	New Zealand	A total of 93 cases per 100,000 servings contained *Campylobacter* spp., 201 cases contained *Shiga toxin*-producing *E. coli* (*STEC*), and 15 cases contained *Salmonella* spp. for *Listeria monocytogenes*.	[45]
2011	*Listeria monocytogenes* *E. coli O157:H7*, *Campylobacter*, *Salmonella*	New York	A quantitative risk assessment in New York estimated *Listeria monocytogenes* infections from raw milk consumption to range from 2.7 × 10^−7^ to 1.0 × 10^−4^ cases per person per year.	[46]
2007–2011	Campylobacter jejuni *E. coli* O157:H7	Italy	Between 2007 and 2011, an estimated 6.3–7.2 cases of HUS (Hemolytic Uremic Syndrome) were linked to raw milk consumption in Italy, caused by *E. coli* O157:H7. Additionally, outbreaks of *Campylobacter jejuni* were reported in the Veneto and Marche regions during the 2008–2009 period, and two *E. coli* O157:H7 outbreaks occurred in Emilia Romagna over the same period.	[47]
2009	*S.aureus Staphylococcus enterotoxin A*	California	A total of 25.3% of 51,963 raw milk samples tested positive for *Staphylococcus aureus*, indicating a substantial contamination rate. Additionally, Staphylococcal Enterotoxin A (SEA) exposure levels at these high percentiles could reach 94 ng/serving.	[48]
2008	*Campylobacter* spp.	California	Of 16 cases, 4 cases were CC for *Campylobacter*; 3/4 drank raw milk; and the rest were employees. Two individuals were hospitalized, including one with a form of Guillain–Barré syndrome.	[49]
2007	*C. jejuni*	Kansas	Of 25 cases, 7 cases were CC, 18 probably occurred over several months; 16/28 persons who consumed raw milk at a gathering became ill.	[50]
2007	*Salmonella typhimurium*	Pennsylvania	There were 29 cases, with an age range of 5 months–76 years; 16/29 were <7 years, 29 cases were CC, there were identical PFGE patterns, and two individuals were hospitalized.	[51]
2007	*C. jejuni*	Kansas	There were 68 cases, and 4 cases were CC for *C. jejuni*; two individuals were hospitalized.	[52]
2006–2007	*Salmonella*		There were 85 cases, primarily including Hispanic people. A total of 85 cases were CC, with identical PFGE patterns; 36 individuals were hospitalized.	[53]
2006	*E. coli O157:H7*	California	There were 6 cases, with 5 CC with identical PFGE patterns. There was one non-CC case, HUS; three individuals were hospitalized.	[54]
2005	*E. coli* O157:H7	Washington	Of 18 cases, 8 cases were CC; 7/8 had identical PFGE patterns. Five people were hospitalized, and four had HUS.	[53]
2002–2003	*S. typhimurium*	Multi-State in the USA	There were 62 cases, and 62 were CC, with identical PFGE patterns and an epidemiologic link to an implicated dairy outbreak strain isolated from milk, cream, and butter samples.	[55]
2002	*C. jejuni*	Utah	Of 13 cases, 5/6 cases were CC; six individuals sought medical attention and none were hospitalized.	[56]
2001	*Salmonella*	-	A total of 26 cases were CC for MDR-SN; 23 individuals were treated with antibiotics, and 8 were hospitalized.	[57]
2001	*C. jejuni*	Wisconsin	Of 75 cases, 28 cases were CC; the PFGE patterns of 21 tested individuals were identical.	[58]

CC: culture confirmed; PFGE: pulsed-field gel electrophoresis, HUS: hemolytic uremic syndrome, MDR: multiple drug resistant.

## 4. Effect of Pasteurization and Sterilization on Milk Constituents

### 4.1. Effects on Milk Protein Structure and Functionality

The heat treatment of milk leads to various protein alterations by denaturation, aggregative interactions, Maillard reactions, and a loss of nutritional value [59]. Different chemical reactions may occur during pasteurization, including denaturation, hydrolysis, glycation, β-elimination reaction, iso-peptide bond formation, and racemization. The milk protein denaturation range by heat falls between 62 °C and 72 °C [60]. The series of reactions during the heating of milk involves different amino acids, especially lysine, tryptophan, asparagine, threonine, phosphoserine, and glutamine, and can affect various milk characteristics. The amount of lysine is reduced by heat treatment, mainly due to Maillard reactions with lactose [61]. Amino acids are building blocks and key components of milk proteins, contributing to nutrition, bioactivity, and functional properties like emulsification and gelling. Heat-sensitive amino acids such as lysine and tryptophan undergo chemical changes during thermal processing, including Maillard reactions and oxidation. These reactions reduce amino acid availability, impair protein digestibility, and affect milk’s flavor, color, and stability [62].

**Figure 3:**
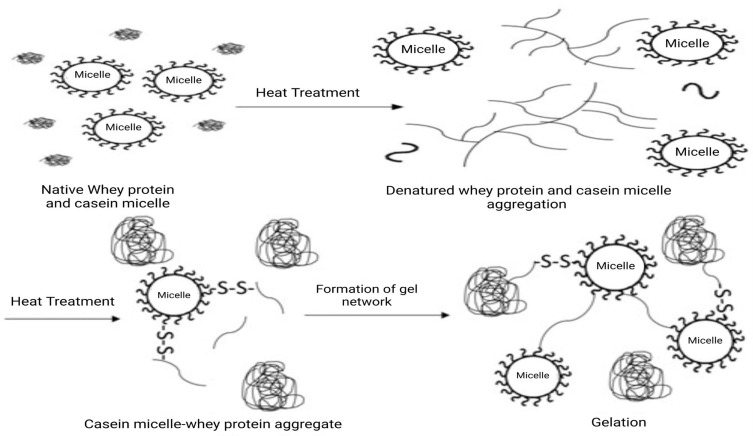
Schematic diagram showing possible interactions in heat-treated milk system. Modified from [66].

Montilla [63] reported that changes in pH at different temperatures caused the denaturation of milk proteins. Partial denaturation of the globular structure of native whey proteins at temperatures above 60 °C causes the unfolding and exposure of their hydrophobic residues and disulfide bonds. Although these reactions may be reversible at low temperatures, new irreversible hydrophobic interactions may be formed by them at high temperatures [64]. Unfolded proteins can also aggregate with other proteins through disulfide linkages and sulphydryl-disulfide interchanges, as shown in Figure 3 [65].

Heating milk above 60 °C may also lead to interactions between the casein micelles and the denatured whey proteins and cause their conversion from a soluble form to a colloidal state [67]. Heated casein micelles increase in size and associate with unfolded whey proteins, leading to the formation of adhesive hard spheres and an increase in viscosity [68]. The association between caseins and whey proteins is caused by hydrophobic interactions at temperatures <70 °C and disulfide bonds at higher temperatures [65]. For example, complexes between β-lactoglobulin and κ-casein aggregates are formed in bovine milk upon heating [65]. Upon heating at low temperatures for an extended time, β-lactoglobulin has enough time to unfold and associate with the micelle. Still, it does not unfold appropriately at high temperatures and may refold into a new structure and form aggregates with molecular species other than κ-casein. The formation of β-lactoglobulin/κ-casein complexes increases with the increased proportion of β-lactoglobulin in milk. Upon prolonged heating at low temperatures, α-lactalbumin forms complexes with β-lactoglobulin and, consequently, with κ-casein (Table 3) [59,69,70].

Some heat-stable indigenous enzymes, such as plasmin and cathepsin, are activated by milk heating, leading to proteolysis during storage [73]. Bovine milk β-casein is hydrolyzed by plasmin (EC 3.4.21.7) to produce three C-terminal fragments [γ1- (fractions 29–209), γ2- (fractions 106–209), and γ3- (fractions 108–209)], αs1-casein is hydrolyzed to produce 14 peptides, and to a lesser extent, αs2-casein is hydrolyzed to release several fragments [74,75,76]. For example, five peptides were identified in heat-treated bovine milk resulting from the enzyme hydrolysis of α_s1_- and β-caseins (Table 4). However, plasmin activity in bovine milk is strongly inhibited by native and denatured β-lactoglobulin [77]. This might explain the lower prevalence of hydrolytic peptides in bovine milk compared to camel milk, which lacks β-lactoglobulin [78]. Plasmin activity is believed to be enhanced by heat treatment and to contribute significantly to age gelation in UHT-treated milk [75].

**Figure 4:**
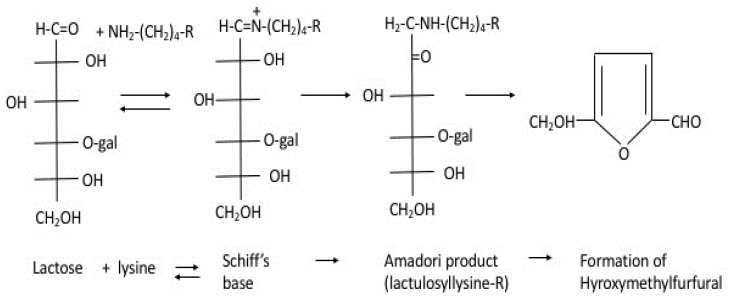
Maillard reaction between lactose and lysine residues in milk.

The Maillard reaction (nonenzymatic glycation) is a chemical reaction between the amino and carbonyl groups. Upon heating, lactose reacts with the ε-amino groups of lysine residues in milk proteins to form the Amadori product (lactulosyl-lysine-R), followed by the elimination of galactose moiety from lactose through the 4-deoxyosone pathway and the formation of an amino-reductone structure [79]. A small reduction (1–4%) in lysine concentration has been observed in pasteurized milk [80]. In the final stages of this reaction, hydroxymethylfurfural and other pigments are formed, causing browning of the milk, especially when heated at high temperatures (Figure 4). Tryptophan is destroyed during pasteurization, and mutagenic derivatives can be formed [81]. Arginine may also be converted into ornithine and citrulline by severe heat treatment, and deamination occurs when excess heat is applied to glutamine and asparagine [82]. These reactions cause a loss of nutritional value in protein because the altered amino acids might become unavailable for the metabolic process or because they cause the formation of toxic end products.

The heat stabilities of milk proteins expressed in terms of Arrhenius kinetics are shown in Table 5. Immunoglobulins and bovine serum albumin are the least stable, while β-lactoglobulin has intermediate stability [59,83]. Because α-lactalbumin is more sensitive to heat than β-lactoglobulin, it denatures at ~62 °C. However, its unfolding is reversible and does not form aggregates at temperatures below 80 °C [69]. Bovine whey proteins are subject to significant alterations at temperatures >130 °C, with β-lactoglobulin experiencing more changes than α-lactalbumin [63].

In conclusion, pasteurization induces a range of structural and chemical changes in milk proteins, including denaturation, aggregation, Maillard reactions, and enzymatic modifications, which can lead to both positive and negative effects. On the one hand, the heat-induced denaturation of whey proteins enhances digestibility and can improve certain functional properties such as viscosity and emulsification. On the other hand, excessive or prolonged heating may result in the loss of essential amino acids (e.g., lysine and tryptophan), a reduced nutritional value, and the formation of potentially harmful by-products. Additionally, heat-activated enzymes like plasmin can contribute to post-pasteurization proteolysis and shelf life challenges such as age gelation. Thus, while pasteurization is vital for ensuring microbial safety, its impact on protein quality is a balance between desirable functional outcomes and potential nutritional drawbacks, which are highly dependent on the processing conditions applied.

### 4.2. Effect on Antimicrobial Systems

While pasteurization is intended to destroy the most heat-tolerant pathogens, it might destroy essential nutrients, enzymes, and microorganisms in milk. Raw milk has been suggested to contain numerous antimicrobial systems that prevent the growth of pathogens and contribute to immunity, including lysozyme, xanthine oxidase, and lactoperoxidase. During storage, the activity of bacteria and other organisms is restricted due to cold or very low temperatures, along with the pasteurization effect [33]. While evaluating the antimicrobial activity of raw milk against pasteurized milk, Pitt [89] found that *Salmonella enteritidis* and *S. aureus* increased in both kinds of milk at 37 °C. However, the level of the pathogen in raw milk decreased after 32 h of growth, suggesting that the inactivation of antimicrobial activity in raw milk was caused by pasteurization. The prevalence of *S. aureus* and *S. enteritidis* after 70–72 h of inoculation in pasteurized milk was almost 100 and 1000 times higher than in raw milk, respectively [33]. *L. monocytogenes* inoculated at 37 °C in raw milk resulted in an initial bacterial population of about 10^4^ CFU/mL after 12 h, after which it lost its ability to grow [90]. No viable cells of *L. monocytogenes* were found 56 h after inoculation in raw milk, suggesting that the microorganism had been “killed” by raw milk. In another study, the impact of unpasteurized milk on the level of *L. monocytogenes* at 15 °C was investigated [91]. During the study of the inhibitory role of the lactoperoxidase mechanism, six different strains of *L. monocytogenes*, which were isolated from unpasteurized milk, were used. The level of *L. monocytogenes* in heat-treated milk was found to increase by 2–3.8 log cycles after 65 h in static conditions [90]. In another study, the population of *L. monocytogenes* in raw milk increased by 0.8–2.3 log cycles under the same conditions [33]. Interest in components of the milk fat globule membrane (MFGM), such as lipid antimicrobial and antiviral properties (e.g., sphingomyelin, phosphatidylcholine, and phosphatidylethanolamine) and the peptide fragments of casein, has been increasing over time [92,93]. Although nearly all antimicrobial agents are destroyed by UHT treatment, their presence is no longer necessary since UHT-treated milk is practically sterile [94].

Raw milk contains various enzymes, many of which are unknown for their biological function or beneficial effects, which are affected by heating (Table 6). There are also some indigenous enzymes in raw milk, and excessive heat treatment can usually destroy and denature beneficial enzymes such as alkaline phosphatase and xanthine oxidase. Alkaline phosphatase (EC 3.1.3.1) indicates an effective pasteurization process due to its inactivation under pasteurization conditions [95]. Xanthine is a milk enzyme that activates lactoperoxidase with the help of hydrogen peroxide. It produces various products that destroy oxidative stress, thus having antimicrobial properties [96]. Enzyme activity is affected by temperature, thermal conductivity, pH, and the presence of substrates, inhibitors, and activators [97].

In summary, while pasteurization enhances milk safety by eliminating pathogens, it also compromises the natural antimicrobial systems inherent in raw milk. Key enzymes and bioactive components such as lactoperoxidase, lysozyme, and xanthine oxidase, known to inhibit pathogen growth, are significantly reduced or inactivated by heat treatment. This inactivation may diminish the milk’s natural defense mechanisms, making it more susceptible to microbial proliferation during storage. Therefore, understanding and preserving these antimicrobial properties, where possible, is crucial for balancing the safety and functional quality of milk.

## 5. The Effects of Pasteurization and Sterilization on the Physical Properties and Sensory Quality of Milk

The sensory quality of milk is defined by its appearance, texture in the mouth, odor, flavor, and taste [108]. During pasteurization, milk undergoes a variety of reactions that might affect its color, flavor, and organoleptic properties, such as the denaturation of proteins, lipid degradation, and Maillard reactions [109,110]. Raw bovine milk is characterized by a yellow color mainly due to its all-*trans*-β-carotene content and small amounts of lutein, zeaxanthin, and β-cryptoxanthin that are associated with the fat globules [111]. Thus, the intensity of the yellow color of milk is determined by the amount of fat and the size of the fat globules, which are also affected by milk standardization and homogenization processes. Heat treatments may destroy carotenoids by oxidation.

The milk viscosity was not significantly affected by mild heat treatments. Still, it increases in severe heat treatments due to changes in protein structures and the formation of larger particles and aggregates. Pasteurization at 60–65 °C causes a slight decrease in viscosity, but pasteurization at 70 °C and higher temperatures may cause significant increases in viscosity [112]. The denaturation of β-lactoglobulin exposes its free sulfhydryl groups, causing its dimerization/oligomerization and aggregation with other whey and casein proteins through sulfhydryl–disulfide interchange reactions [113]. These interactions may lead to the formation of weak three-dimensional structures with liquid-like behavior, contributing to increased viscosity [113]. Age gelation occurs during the storage of UHT-treated milk (~>12 weeks at 20–25 °C) through the formation of a robust extended solid-like gel [114]. The viscosity of mildly processed milk increases with an increased fat content and the presence of more and larger fat globules, which increase the resistance of milk to flow [115,116].

Heat treatment significantly influences the flavor profile of milk by promoting chemical changes such as protein denaturation, Maillard reactions, and lipid oxidation. These reactions are especially prominent in ultra-high-temperature (UHT) processing and ultra-pasteurization, which exceed 90 °C. One of the effects of heat is the unfolding of whey proteins, such as β-lactoglobulin, which leads to the release of volatile sulfur compounds like hydrogen sulfide (H_2_S), methional, and dimethyl sulfide, which in turn causes a cooked flavor in milk [117]. UHT treatment causes ketone and a cooked flavor in milk due to the presence of methyl ketone and the oxidation of sulfur and lactone compounds, which originate in the lipid part of milk. This flavor remains in milk for some time and may disappear within a week depending mainly on the type of UHT process applied. Flavor compounds, such as sulfur and nitrogen-containing compounds, diacetyl, Strecker aldehydes, and maltol, are produced during thermal processing when amino acids and lactose in milk undergo Maillard reactions [118]. Methyl ketone is generated due to the β-oxidation of free fatty acids, which is induced by the degradation of lipids during pasteurization [119]. Ultra-pasteurized milk is characterized by its various distinct flavors, such as cooked, caramelized, and sulfurous flavors, which make it different from HTST milk. These distinctive flavors are a drawback of ultra-pasteurized milk [120]. Due to the presence of sulfur-containing compounds (e.g., dimethyl sulfide, hydrogen sulfide, methional, carbon disulfide, dimethyl trisulfide, and Maillard compounds, i.e., furfural, benzaldehyde, 2- and 3-methylbutanal, and 2-acetyl-1-pyrroline), ultra-pasteurized milk is different from HTST milk, which also has distinctive sulfur and cooked flavors. The amount of various sulfur and Maillard-reacting compounds is also influenced by the fat concentration in milk, which, in turn, affects the flavor and taste of milk [118].

Zhao [121] demonstrated that HTST milk (63 °C/30 min or 72 °C/15 s) contains higher levels of low-molecular-weight compounds like 2-butanone and dimethyl ketone, whereas UHT processing using Direct Steam Injection (DSI) at 150 °C for 0.1 s leads to a notable rise in high-molecular-weight aldehydes such as hexanal, pentanal, and nonanal. These aldehydes contribute to grassy, fruity, and floral notes in UHT-treated milk in contrast to the lighter, less complex flavor of HTST milk. Benzaldehyde, an aromatic compound from the Maillard reaction, adds sweet almond-like notes, while dimethyl sulfone is primarily linked to sulfurous off-flavors [122]. Consumer preferences vary widely; Liem [123] reported that in China, where 60% of the population consumes long-life milk, the cooked and sulfurous flavors are better accepted, whereas Australian consumers, who primarily consume fresh HTST milk, prefer milder flavors, highlighting regional differences in flavor tolerance and product expectations.

Meng [124] further evaluated DSI and DSIJ (Direct Steam Injection Jet) treatments using the solvent-assisted flavor evaporation (SAFE) and solid-phase microextraction (SPME) techniques. They identified 59 volatile compounds across both methods, including nonanal, 2-undecanone, 2-decanone, δ-decanolactone, 3-hydroxy-2-butanone, and dimethyl sulfone. SAFE favored the detection of alcohols and aldehydes, while SPME excelled in extracting esters and sulfur-containing volatiles. DSIJ milk exhibited slightly more diverse compounds (52) compared to DSI milk (50), suggesting subtle differences in flavor complexity based on thermal intensity and duration. Ultimately, while UHT-treated milk is thermally stable and has an extended shelf life, its cooked, caramelized, and sulfur-rich profile remains a sensory drawback in markets favoring a fresh dairy flavor. The fat content and storage time further modulate these effects by enhancing the concentration of thermally derived volatiles over time.

In summary, pasteurization influences the physical properties and sensory quality of milk through various biochemical and structural changes. While mild heat treatments have a minimal impact on viscosity, higher temperatures can lead to protein denaturation, increased viscosity, and gel formation over time. Additionally, thermal processing induces flavor alterations, including the development of cooked, caramelized, and sulfurous notes, which are more pronounced in ultra-pasteurized milk. Despite these changes, pasteurization remains essential for ensuring milk safety and quality.

## 6. The Effects of Pasteurization and Sterilization on the Nutritional Quality of Milk

### 6.1. Effects on Vitamins and Minerals

Milk has a balanced nutritional value and digestible elements necessary for the development of babies. About 87% of milk is water, and the remaining 13% constitutes nutritionally valuable components, including carbohydrates, minerals, proteins, lipids, and vitamins. Milk is also one of the best sources of essential amino acids, calcium, phosphorous, and high-quality proteins that contribute to human nutrition, reproduction, growth, and the promotion of the development of bones and muscles. The increase in the global consumption of milk and milk products often underlines the critical question of how different pasteurization processes may affect the nutritional constituents of raw milk. Different pasteurization conditions may influence the physicochemical characteristics of milk and milk products differently. The pasteurization process decreases the amount of total fat in raw milk. For example, the total fat in raw milk was found to decrease from 3.58% to 3.07% after pasteurization and to decrease further after UHT treatment [125]. However, the fat contents of milk sold on the market are standardized to 3.5% in full-fat milk, 1.5–1.8% in semi-skimmed milk, and 0.5% in skimmed milk by adding or removing cream (Council Regulation (EC) 2597/97) [126].

Fat- and water-soluble vitamins are destroyed by excessive heat treatment or pasteurization. A drastic reduction in the levels of some essential vitamins due to pasteurization has been observed in an evaluation of the effect of pasteurization on different vitamins (e.g., A, B1, B2, B6, B12, C, and E) in a meta-analysis based on 40 different studies [127]. It was observed that pasteurization leads to some decline in the levels of vitamins, especially vitamin B2 and folate. Minor differences between raw and pasteurized milk were observed in thiamine (vitamin B1) and pyridoxine (vitamin B6) concentrations. However, the concentration of vitamin A was found to increase after pasteurization [127]. Minerals are generally heat-stable during pasteurization [128]. Pasteurization does not seem to affect the bioavailability of milk calcium and phosphorous [129]. In conclusion, pasteurization can lead to moderate losses of certain heat-sensitive vitamins, particularly B-group vitamins and vitamin C. However, essential minerals like calcium and phosphorus remain largely unaffected, preserving milk’s core nutritional value. Despite minor nutrient reductions, pasteurized milk continues to serve as a vital source of high-quality proteins, essential vitamins, and minerals, supporting human growth and development across all age groups.

### 6.2. Effect on Milk Digestibility and Gut Health

Bovine milk is naturally rich in psychotropic lactic acid bacteria (*Lactobacillus*, *Lactococcus*, *Leuconostoc*, *Streptococcus*, *Enterococcus*, and small proportions of *Acinetobacter* and *Pseudomonas*), which cause spoilage in milk. The importance of the gut microbiome can influence psychological functioning and affect different aspects of mental as well as physical health. The composition of the gut microbiome mainly depends on the diet of the person. Enhancing growth and maintaining healthy gut microbiota in the diet is challenging. It was demonstrated in epidemiological studies that ingesting raw milk enhances the growth and composition of the gut microbiome [130]. Researchers evaluated the outcome of different dietary intakes on the gut microbiomes of several people who went through a 12-week research course on a farm and consumed unpasteurized milk and dairy products produced from a herd of Jersey cows. Most people who participated in this study did not consume unpasteurized dairy products before this course. The study concluded that there was a significant change in the microbiome, with *Lactobacilli* levels dramatically increasing in the fecal samples from pre-course to post-course [131]. The consumption of unpasteurized milk and dairy products was identified as the main reason for the increase in the levels of the gut microbiome. This increase is beneficial because *Lactobacilli* are known to support gut health by improving digestion, enhancing immune function, and inhibiting the growth of harmful bacteria.

### 6.3. Effects on Lactose Intolerance, Allergy, and Immunity

Lactose, the predominant carbohydrate available in mammalian milk, has several health benefits, including energy production, low glycemic levels, facilitation of the absorption of magnesium and calcium, and prebiotic properties, especially for children [132]. The enzyme lactase (β-galactosidase) is responsible for the digestion of lactose in humans. The absence of this enzyme results in lactose intolerance manifested as symptoms, including bloating, diarrhea, flatulence, and severe abdominal pain. More than 65% of the human population is projected to suffer from lactose intolerance, for which the prevalence increases with age [133]. There are widespread anecdotal claims that raw milk has a curing effect on lactose intolerance due to its content of natural lactase, and bacteria that produce this enzyme, mainly *Lactobacillus acidophilus*, are destroyed by pasteurization [127]. Still, this hypothesis was later rejected [134]. However, there is no evidence supporting these claims. In a recent pilot cross-over intervention study, it was shown that raw milk was not different from pasteurized milk in affecting the symptoms of lactose malabsorption or intolerance in adults suffering from lactose malabsorption [135].

The increase in allergies and asthma in Western countries was associated with the consumption of pasteurized milk [136]. In this case, there is consistent evidence that children raised on farms show decreased incidences of allergic hypersensitivities, hay fever, atopy, and asthma compared to other children. However, the exact reason(s) cannot be given and may be multifactorial [137,138,139,140,141,142,143,144,145,146]. Rosenlund [147] collected data that included about 15,000 children from five European countries with different lifestyles. It was found that children living in agricultural areas and with restricted use of vaccines, antibiotics, and antipyretics showed a negative association between unpasteurized milk consumption and asthma and allergies (Table 7). In another study including about 1,000 rural children from five European countries, Loss [148] found a negative relationship between raw milk consumption and rhinitis, otitis, and respiratory tract infection. In the hygiene hypothesis, Strachan [149] suggested that farm living and related lifestyles expose children to microbes and may protect them against some allergies. However, in a study of germ-free female BALB/cByJ mice, it was shown that the pasteurization of bovine milk improves the allergenicity of β-lactoglobulin by denaturation and suppression of the effect on conformational, i.e., sequential epitopes [150].

Pasteurization and heat treatment are believed to destroy the beneficial immunoglobulins naturally present in raw milk, although this concentration is too low to have any physiological significance in human immunity [156]. Mainer [157] concluded that there was no effect on the IgG level upon LTLT pasteurization and only a 1% reduction upon HTST pasteurization. In another study, Kulczycki, [158] reported that heat-induced aggregation could be enhanced by pasteurization, increasing the receptor-binding activity of immunoglobulin IgG. This means that pasteurized milk has better immunological function than raw milk. A potent immune-regulatory molecule known as transforming growth factor-β (TGF-β) is found in raw milk, and it has been reported that pasteurization has no effect on it [159]. In raw milk, the concentrations of immune-modulatory factors, including TNF-α, IL-1β, IL-10, and IL-6, are too small to induce any physiological significance [160]. However, there is a hypothesis that higher immunity against symptomatic infections caused by pathogens can be achieved by frequent consumption of raw milk. This happens because of continuous exposure to the nonvirulent strain of the pathogen, which can develop cross-immunity. However, the only case reported in the literature is specifically with *Campylobacter* [161].

Products from the Maillard reaction have both beneficial and harmful effects on health. Depending on how the food is prepared or processed, toxic and beneficial Maillard reaction products can be formed. Diverse Maillard reaction products can act as anti-browning, antioxidant, prooxidant, bactericidal, carcinogenic, and anti-allergic agents. The majority of these characteristics are influenced by different food processing techniques. Due to pasteurization or high-temperature processing, some foods can lose their nutritional content, while others become more nutritious. Several measures may be applied in the food industry to limit or reduce the generation of Maillard reaction products. For example, acrylamide produced at high temperatures was classified as a possible human carcinogen (International Agency for Research on Cancer, 1987) [162]. Asparaginase has been effectively used in the laboratory to decrease acrylamide. Injecting carbon dioxide during the extrusion process can also help lower acrylamide levels [163]. Maillard reactions may form other compounds, such as furosine, lactulose, HMF, etc., that can negatively affect long-term health. Finally, there is concern that milk exosomes are affected negatively by pasteurization and that human exposure to these exosomes may induce risks of developing chronic diseases, including obesity, type 2 diabetes mellitus, osteoporosis, cancers, and Parkinson’s disease [164,165].

While pasteurization has a minimal effect on lactose intolerance and may slightly alter allergenicity and immune factors, current evidence does not support claims that raw milk improves these conditions. Although some bioactive compounds and enzymes are reduced, pasteurized milk maintains nutritional safety and may even enhance certain immunological functions. Moreover, the loss of natural components is often outweighed by the reduction in foodborne illness risk.

### 6.4. Effect on Fatty Acids and Milk Fat Globule Membrane (MFGM)

Milk fat content in commercial products is typically regulated through standardization, achieved by adjusting the cream content to produce full-fat, semi-skimmed, or skimmed milk. However, heat treatments, particularly at high temperatures, can induce both physical and chemical transformations in the milk lipid fraction. Notably, these changes include an increase in free fatty acid levels. Moreover, polyunsaturated fatty acids in milk are susceptible to thermal alterations, including the formation of conjugated isomers under severe heat treatment. One such compound, conjugated linoleic acid, is recognized for its potential anti-carcinogenic effects, indicating that some thermal transformations may have beneficial implications [166]. Nevertheless, the impact of heat on the overall fatty acid profile appears limited under typical pasteurization conditions. A study by Pestana [125] found no significant changes in the total fat content or overall fatty acid composition following pasteurization, suggesting that milk lipids exhibit relative resilience to moderate heat processing.

At the same time, pasteurization disrupts the structural integrity of the milk fat globule membrane (MFGM), a bioactive interface rich in phospholipids and glycoproteins. Damage to the MFGM may reduce lipid protection against oxidation and alter fat digestibility [167]. Additionally, the loss of MFGM-associated components such as sphingomyelin could have implications for infant brain development and immune function [168]. These findings emphasize the need to consider not just fat quantity but also structural and functional aspects of milk lipids in processing evaluations.

## 7. Alternative Processing Methods

High-pressure processing (HPP), power ultrasonics (PU), and pulsed electric fields (PEFs) are non-thermal processing technologies with promising impacts on food processing [169]. HPP is a substitute for conventional thermal treatment methods for foods, and it has been shown to better preserve the nutrients and bioactive components in milk [170]. The key benefits of high HPP are the retention of sensory properties and the nutritional value of food by the inactivation of microorganisms. Some commercially available pressurized foods include yogurt, fruit jams, juices, avocado pulp, jellies, and sauces [171,172]. Batch or semi-continuous equipment is used to conduct treatments, usually in the range of 100–1000 MPa, for up to 30 min to inactivate the spore of the microorganism [173]. HPP parameters include the process pressure, pressure hold time, initial product temperature, time to achieve pressure, treatment temperature, water activity, pH of the product, temperature distribution in the vessel, and decompression time, among others [174]. Under the influence of HPP, nucleic acids, enzymes, polysaccharides, and proteins may be affected, but due to their simple structure and small molecular size, vitamins and amino acids remain unaffected [173].

Hite [175] studied the effect of HPP on milk bacteria and concluded that processing at 680 MPa for 10 min would lead to complete sterilization and delay the microbial spoilage of milk for seven days. The inactivation of microorganisms by HPP involves several mechanisms, such as membrane destruction, changes in bacterial enzymes and nucleic acids, and simultaneous leakage of the contents of organelles and cells [176]. Factors like the composition and pH of food, state and type of growth, and time and pressure of application can affect the efficiency of HPP, while the effect of HPP on enzymes in milk is comparatively much less than that of heat. Several researchers examined the impact of HPP on endogenous enzymes in milk, such as glutamyltransferase, alkaline phosphatase, phosphohexose isomerase, and lactoperoxidase [172,177]. The inactivation of plasmin by HPP requires a pressure of up to 600 MPa and a temperature of more than 20 °C [178,179]. It has been shown that whey protein (mainly β-lactoglobulin) and caseins are altered by HPP [178,179]. The denaturation of β-lactoglobulin occurs at a pressure of more than 100 MPa at 25 °C, while bovine serum albumin and α-lactalbumin tolerate pressures around 400 MPa without being denatured [179]. Studies show that HPP at 600 MPa for 3 min can achieve a 5-log reduction in *Listeria monocytogenes* and *E. coli*, similar to thermal pasteurization, but without the associated thermal degradation of bioactive compounds [180]. Quantitatively, HPP-treated milk retains up to 90% of vitamins B2 and C, whereas conventional pasteurization retains only around 50–60% [181]. Over the past 20 years, HPP technology has advanced dramatically, but its high installation cost and the complexity of designing a continuous system to prevent corrosion and cross-contamination are drawbacks in the industrial application of HPP for milk treatment.

Ultrasound waves, which can pass through milk at a frequency greater than 20 kHz, in milk processing may offer advantages to the dairy industry, such as improved product quality and cost-saving properties. However, ultrasound alone is not very efficient in deactivating microorganisms and enzymes; therefore, it should be combined with sonication techniques [174,182]. Ultrasound treatments are affected by several factors, including enzyme concentration, medium composition, treatment level, frequency, and energy density [183]. The inactivation of enzymes usually increases with increasing ultrasonic power, temperature, frequency, pressure, exposure time, and amplitude and decreases as the volume of the sample increases [184]. It has been reported that enzyme deactivation decreases with an increase in enzyme concentration and increases with a rise in solid waste [185]. There was no effect on enzymes in milk when ultrasound was applied without heat treatment. Studies demonstrated that sonication at 20 kHz achieved a 5-log reduction in *Escherichia coli* O157:H7 counts in liquid foods like milk and juices, aligning with FDA pasteurization standards [183]. Unlike conventional pasteurization methods, which often reduce heat-sensitive nutrients such as vitamins B1 and C by 20–50%, ultrasound preserves up to 90% of these compounds [186]. This not only improves nutritional quality but also enhances sensory properties and energy efficiency, making ultrasound a viable, sustainable alternative to traditional pasteurization.

Power ultrasonic (PU) milk treatment is an alternative to conventional thermal techniques and has more benefits than pasteurization, e.g., energy usage reduction, the potential to target particular species, and the absence of preservative requirements [185]. The deactivation of bacteria and enzymes, milk homogenization, lactose hydrolysis, and the extraction of enzymes are the main applications of PU in the dairy industry. Expansion cycles and alternating compression are formed when ultrasonic waves pass through the liquid, causing the growth of existing bubbles by high-intensity ultrasound in the expansion cycle, which violently implode when they reach a volume at which more energy is not absorbed, a process called cavitation [187]. Physical forces created by acoustic cavitation are the primary mechanism responsible for ultrasonic microbial deactivation.

The main criteria for evaluating any new technology to replace thermal treatment methods are its ability to provide the consumer with a safe, shelf-stable, cost-effective, and better-quality product. Using a pulsed electric field (PEF) could be a suitable alternative to traditional heat or thermal treatments for different liquid and semi-liquid foods as it can destroy harmful microorganisms and some enzymes while retaining the quality and freshness of food products. The application of PEF technology to pasteurize food, such as yogurt, juices, soups, liquid eggs, and milk, has been successfully demonstrated with negligible effects on the nutritional and sensory quality of food [188]. The inactivation of microbes and enzymes by PEF depends on different parameters, such as composition, ionic strength, pH, and conductivity [189]. PEF induces minimal deactivation of enzymes and bacterial spores, so it needs to be combined with other technologies, such as thermal treatment and the addition of bacteriocins or antimicrobial agents. It has been observed that PEF treatment is successful in destroying vegetative microorganisms, but the high deactivation of spoilage enzymes and microbial spores requires other combined treatments. The minimal effect on enzymes benefits the dairy industry as milk can be processed to inactivate microorganisms while retaining beneficial enzyme activities [190]. PEF processing of unpasteurized milk has been reported to have a minimal effect on the tertiary structure of whey proteins [191]; for example, the folded structure of heat-sensitive lactoferrin was retained after PEF processing regardless of the processing mode. PEF does not affect the sensory, physical, or chemical properties of milk [192,193]. Compared to traditional thermal pasteurization, PEF has demonstrated concrete microbiological and nutritional advantages. Quantitatively, PEF-treated milk and juice samples have shown microbial reductions ranging from 0.13 to 6.2 log CFU/mL for total bacteria and up to a 0.48 log reduction in coliforms depending on the treatment parameters and food matrix [194]. While traditional pasteurization may cause significant degradation of heat-sensitive nutrients and flavor compounds, studies confirmed that PEF causes negligible nutrient loss and leads to better preservation of antioxidant and sensory qualities [195]. PEF treatments provide less destruction of flavor and nutrients and retain the quality and nutritional value of processed foods. Further research is needed to attain a higher inactivation rate and to study more factors affecting PEF treatment to make it more precise.

Microfiltration (MF) is a non-thermal processing technology increasingly utilized in the dairy industry as an alternative to traditional heat pasteurization. This membrane-based technique relies on porous filters, typically with pore sizes ranging from 0.1 to 1.4 microns, to physically separate microorganisms, including bacterial spores, from milk without significantly altering its biochemical composition [196,197]. The key benefit of MF lies in its ability to retain milk’s native organoleptic properties, such as its taste, aroma, and texture, while extending the shelf life, making it highly attractive for premium milk products [198]. Unlike thermal pasteurization, MF avoids protein denaturation and the loss of heat-sensitive nutrients, thereby preserving nutritional quality. This approach also enables selective component separation, for example, removing somatic cells or bacteria while allowing fat and casein micelles to pass, offering functional advantages in cheese manufacturing and protein standardization [199]. However, membrane fouling is a persistent limitation. The accumulation of proteins and fats on the membrane surface can reduce filtration efficiency and require frequent cleaning cycles, thus impacting the operational cost-effectiveness of the system. Moreover, emerging evidence suggests that certain spoilage microbes may still survive or proliferate post-filtration, highlighting the need for complementary treatments like UV or high-pressure processing in some applications [200]. Despite these challenges, MF remains a promising technique, offering flexibility, product quality enhancement, and potential for integration into hybrid preservation systems for next-generation dairy products.

## 8. Economic Impact of Milk Processing Methods

The choice of milk processing technology significantly influences both the economic viability and sustainability of dairy enterprises. Traditional thermal pasteurization methods, although well established, are energy-intensive and may result in higher operational costs due to prolonged heating and cooling cycles, labor, and maintenance requirements. In contrast, alternative processing methods such as high-pressure processing (HPP), pulsed electric field (PEF), and ultrasound-assisted pasteurization and microfiltration offer promising avenues for cost-efficiency and product quality retention.

However, their implementation carries varying economic implications. A study evaluating alternative dairy processing models revealed that small-scale facilities (50–500 cows) for fluid milk, yogurt, and cheese require capital investments ranging from USD 1.5 million to USD 7 million depending on product line and automation levels. Operational costs, including energy, labor, and packaging, represent a significant portion of the total expenditure, with energy savings being a potential advantage of non-thermal technologies [201].

For example, the adoption of PEF in milk processing, while requiring an upfront capital investment in specialized equipment, can lead to lower processing temperatures and shorter treatment times, translating into long-term energy savings and reduced product loss. This aligns with findings that modern processing methods can enhance efficiency and reduce waste, improving overall profitability [202]. Furthermore, an environmental and economic analysis estimated that traditional fluid milk processing contributes about 2.4 kg CO_2e_ per kg of milk produced and processed, a figure that could be reduced with energy-efficient alternatives like PEF or HPP [203]. Market-dependent variables such as raw milk price, product demand, and consumer preferences also influence the economic feasibility of processing methods. Enterprises must also consider maintenance costs, operator training, and regulatory compliance when adopting novel technologies [204]. In conclusion, while alternative milk processing technologies can offer operational and product quality advantages, careful cost–benefit analyses are essential for industrial application. Decision-makers in the dairy sector should weigh capital investment, energy efficiency, regulatory requirements, and market positioning when considering a transition from conventional to innovative milk processing methods.

## 9. Future Prospective

The future of milk processing technologies lies in achieving a balance between microbial safety and the preservation of nutritional and sensory quality. While conventional heat pasteurization and sterilization have played a major role in public health, consumer demand for minimally processed and nutrient-retentive foods is accelerating the transition toward non-thermal technologies. Emerging innovations such as high-pressure processing (HPP), pulsed electric fields (PEFs), ultrasound, and microfiltration (MF)-assisted non-thermal processing have shown promising results in maintaining milk’s natural bioactives, proteins, and enzymes while ensuring microbial safety. These methods offer energy-efficient and sustainable alternatives that minimize the thermal degradation of heat-labile nutrients like vitamins B1 and B2 and bioactive peptides.

Moreover, recent insights into the gut microbiome have emphasized the need to preserve milk’s native microflora and immunomodulatory compounds. Functional milk enriched with probiotics, prebiotics, and immune-supporting molecules like lactoferrin and transforming growth factor-β (TGF-β) may play a future role in preventing allergies and promoting gut health. Future research may also explore nanoencapsulation and milk exosome protection strategies to safeguard these components during processing. Another promising direction is precision pasteurization, where real-time sensors and AI-driven modeling optimize thermal input to ensure pathogen death while minimizing protein denaturation and flavor loss. Coupled with smart packaging and cold-chain blockchain systems, this could significantly improve the traceability and shelf life prediction of pasteurized milk products. In the coming years, interdisciplinary efforts will be critical for designing next-generation milk processing systems. These should fulfill dual objectives, namely enhanced safety and superior quality, aligning with the principles of personalized nutrition, sustainability, and consumer health consciousness.

## 10. Concluding Remarks

It is suggested by current research that milk pasteurization by heat treatment is adequate to ensure microbial safety but may impact the nutritional or sensory quality of the milk depending on the treatment temperature and time. The consumption of raw milk cannot be encouraged due to the safety risks associated with the possible content of pathogenic microorganisms. More studies that evaluate the differences between raw and differently pasteurized/sterilized milk samples and their relevance to milk quality are required. In addition, the development of alternative pasteurization methods that ensure hygienic collection and assessing the microbial quality of raw milk from individual animals before its inclusion in collection tanks are required. Studies on the digestibility of different kinds of heat-treated milk and their relation to gut health, intolerance, and allergy are also highly warranted.

## Figures and Tables

**Table 3:** Free sulfhydryl (SH) group and disulfide (S-S) bonds in milk whey proteins.

Protein	-SH Groups	S-S Bonds	Reference
α-LA	None	4 (Cys6-Cys120, Cys28-Cys111, Cys61-Cys77, and Cys73-Cys91)	[71]
β-LG	1 (Cys121)	2 (Cys66-Cys160 and Cys106-Cys119)	[72]
BSA	1 (Cys34)	17	[65]

**Table 4:** Peptides generated from bovine α_s1_- and β-caseins after heat treatment [73].

Precursor	Peptide Sequence (Position)	Peptide (*m*/*z*)	Released by
αS1-Casein	IPNPIGSENSEKTTMPLW (182–199)	2014.0	Heat
	SDIPNPIGSENSEKTTMPLW (180–199)	2216.1	Cathepsin G
	RPKHPIKHQGLPQEVLNENLLRFF (1–24)	2910.6	Cathepsin B, Cathepsin D
β-Casein	EMPFPKYPVEPFTESQSL (108–125)	2126.0	Plasmin, Cathepsin D
	HKEMPFPKYPVEPFTESQSL (106–125)	2391.2	Plasmin, Cathepsin D

**Table 5:** Arrhenius kinetics describing the heat-induced degradation of milk whey proteins and lysine.

Component	Temperature Range ( °C)	ln k_0_	E_a_ (kJmol^−l^)	*n*	Reference
Bovine serum albumin	82–150	13.18	49	1	[84]
Immunoglobulin	60–76	90.38	275	1	[85]
	76–82	54.21	170	1	
α-Lactalbumin	70–85	84.92	269	1	[86]
	85–150	16.95	69	1	
β-Lactoglobulin	70–90	89.43	280	1.5	[86,87]
	90–150	12.66	48	1.5	
	75–85	120.64	374	1.8	
Lysine (AA)	130–160	8.77	109	2	[88]

**Table 6:** The effect of pasteurization on antimicrobial milk proteins and enzymes.

Component	Role in Milk	Effect of Pasteurization	References
Alkaline Phosphatase (EC 3.1.3.1)	Potent anti-inflammatory enzyme	Since this enzyme is destroyed by heat, it is used as sensitive indicator for adequate pasteurization of milk	[98]
Bovine immunoglobulin	Immunogenic proteins	59–76% of activity is retained after HTST treatment	[99]
Bacteriocins	Antimicrobial peptides produced by certain milk bacteria with narrow spectrum of antimicrobial activity mainly against Gram-positive bacteria	No effect	[100,101]
Lactoferrin	Broad-spectrum antibacterial agent that binds to iron and reduces free iron supply for proliferation of bacteria, fungi, and protozoa	No effect	[102]
Lactoperoxidase(LPO, E.C. 1.11.1.7)	Acts together with hydrogen peroxide and thiocyanate ions as antibacterial agents	70–90% of enzyme activity is retained after HTST treatment; activity is gradually lost during refrigeration of pasteurized milk	[103,104]
Lysozyme	Breaks cell walls primarily affecting Gram-positive bacteria	>75% of enzyme activity is retained after heating (80 °C, 15 s)	[105]
Plasmin (EC 3.4.21.7)	Milk protease causes alterations in protein structure and function	Survives pasteurization but may be destroyed at high temperature	[106]
Xanthine oxidase	Claimed to have antimicrobial properties by supplying hydrogen peroxidetolactoperoxidase	No effect	[96,107]

**Table 7:** Studies evaluating the effect of raw milk consumption on asthma and allergic diseases.

County Where Study Was Conducted	Exposure	Results	Reference
Crete (Greece)	Unpasteurized milk products	Adj. OR (and 95% CI) of atopy and unpasteurized farm milk consumption with and without simultaneous farm animal contact: 0.32 (0.13–0.78) and 0.58 (0.34–0.98), respectively	[151]
Austria, Germany, Switzerland	Milk directly produced or purchased on a farm	Consumption of farm milk during first year of life significantly inversely associated with asthma, hayfever, and atopy independent of other farm exposures	[141]
New Zealand	Unpasteurized milk, yogurt at least weekly before age of two years	Adj. OR and (95% CI) for early yogurt consumption and hay fever 0.30 (0.1–0.7); any unpasteurized milk and atopic eczema: 0.2 (0.1–0.8); no significant association between unpasteurized milk consumption and asthma or atopy	[146]
Finland	Farm milk in infancy	Farm milk consumption not associated with atopy; no other allergic health outcomes reported	[152]
Northern Germany	Raw, unboiled farm milk	Raw milk consumption and atopy adj. OR (and 95% CI): 0.65 (0.36–1.18); in those visiting animal houses before age of 7 years, raw milk consumption and atopy: 0.35 (0.17–0.74)	[153]
England	Unpasteurized milk	Current unpasteurized milk consumption associated with less eczema adj. Or and (95% CI) of 0.59 (0.40–0.87) and atopy of 0.42 (0.10–0.53), and higher production of whole blood stimulated IFN-γ; effect independent of farming status; no effect on asthma	[140]
Sweden, Austria, the Netherlands, Germany, Switzerland	Milk directly produced or purchased on a farm	Association between farm milk and asthma varied between genotypes of CD14/-1721; similar patterns for symptoms of hay fever and pollen sensitization; CD14/-1721 also modified association between farm milk and CD14 gene expression	[154]
Sweden, the Netherlands, Austria, Germany, Switzerland	Milk directly produced or purchased on a farm	Adj. OR and (95%CI) of farm milk consumption ever in life and asthma: 0.47 (0.61–0.88), rhinoconjunctivitis: 0.56 (0.43–0.73), sensitization to pollen: 0.67 (0.47–0.96), and food mix: 0.42 (0.19–0.92); association observed in all subgroups independent of farm-related exposures	[145]
Finland, France Austria, Germany, Switzerland	Skimmed and unskimmed farm milk, farm-produced butter, and yogurt during pregnancy	Maternal consumption of farm-produced butter during pregnancy associated with increased IFN-γ and TNF-α production in cord blood, and farm-produced yogurt inversely related to these cytokines	[155]

Adj. OR: prevalence and adjusted odds ratio; CI: confidence interval; IFN-γ: interferon gamma; CD14: cluster of differentiation 14; TNF-α: tumor necrosis factor alpha.

## Data Availability

No new data were created or analyzed in this study. Data sharing is not applicable to this article.
